# Long non-coding RNA RP11-284F21.9 functions as a ceRNA regulating PPWD1 by competitively binding to miR-769-3p in cervical carcinoma

**DOI:** 10.1042/BSR20200784

**Published:** 2020-09-28

**Authors:** Hong-Fang Han, Qian Chen, Wen-Wei Zhao

**Affiliations:** 1 Department of Obstetrics and Gynecology, The Second Affiliated Hospital of Xi’an Jiaotong University, Xi’an 710004, China; 2 Department of Dermatology, The Second Affiliated Hospital of Xi’an Jiaotong University, Xi’an 710004, China

**Keywords:** cervical carcinoma, miR-769-3p, PPWD1, RP11-284F21.9

## Abstract

Cervical carcinoma is the most common gynecological cancer in women worldwide. Emerging evidence has shown that long non-coding RNAs (lncRNAs) participate in multiple biological processes of cervical carcinoma tumorigenesis. We aimed to investigate the function of a novel lncRNA RP11-284F21.9 in cervical carcinoma. We found that RP11-284F21.9 was down-regulated in cervical carcinoma tissues and cell lines. Overexpression of RP11-284F21.9 inhibits proliferation, invasion and migration of cervical carcinoma cells *in vitro*. Further, we identified that RP11-284F21.9 directly interacted with miR-769-3p and functioned as the miR-769-3p sponge. Mechanistically, we showed that miR-769-3p regulated peptidylprolyl isomerase domain and WD repeat-containing protein1 (PPWD1) expression by targeting PPWD1 3′-UTR. Furthermore, xenograft tumor model revealed that overexpression of RP11-284F21.9 inhibited tumor growth of cervical carcinoma *in vivo*. Taken together, our results demonstrate that RP11-284F21.9 functions as tumor suppressor and regulates PPWD1 expression through competitively binding to miR-769-3p in cervical carcinoma, suggesting that RP11-284F21.9/miR-769-3p/PPWD1 axis could serve as a promising prognostic biomarker and therapeutic target for cervical carcinoma.

## Introduction

Cervical carcinoma is one of the most common aggressive gynecological cancers and ranks fourth as the leading cause of cancer-related deaths in women worldwide [1]. Significant advances in diagnosis and therapeutic technologies have greatly improved the cervical carcinoma treatment [2]. However, due to the highly invasive and metastatic ability of cervical carcinoma, the prognosis remains poor in patients at advanced cancer stages [3,4]. Thus, it is critical to understand the mechanisms underlying the tumorigenesis and metastasis of cervical carcinoma and develop new therapeutic strategies to treat the disease.

LncRNAs are a class of long non-coding RNAs with >200 nucleotides involved in multiple biological processes including cancer development and metastasis [5–7]. Emerging evidence has shown that various lncRNAs are involved in the regulation of cervical carcinoma tumorigenesis and functions as oncogenes or tumor suppressor [8]. For instance, lncRNA AB073614, SNHG14, DANCR and MNX1-AS1 promote cervical carcinoma progression via different signaling pathways [9–13]. Other lncRNAs, such as WT1-AS and TUSC8 inhibit the proliferation, migration and invasion of cervical cancer cells [14,15]. RP11-284F21.9 is a new lncRNA defined by pan-cancer transcriptomic analysis [16]. However, the function of RP11-284F21.9 in cervical cancer is not clear.

MiRNAs are small non-coding RNAs with ∼22 nucleotides that post-transcriptionally regulate gene expression by binding to the 3′-UTR of their target mRNAs [17]. Increasing studies have found that miRNAs play critical roles in the tumorigenesis of most human malignancies, including cervical cancer [18–20]. Interestingly, lncRNAs can exert their functions as competing endogenous RNAs (ceRNAs) and sponge activity for miRNAs [21–23]. Multiple studies have revealed the lncRNA–miRNA–mRNA interaction network in cervical carcinoma [24,25]. LncRNA SOX21-AS1 could sponge miR-7/VDAC1 and promote cervical cancer development [26]. Wang et al. reported that lncRNA NOC2L-4.1 regulated miR-630/YAP1 pathway and functioned as a tumor oncogene in cervical cancer [27]. Though much progress has been made, the understanding of the roles of lncRNA–miRNA–mRNA network in cervical carcinoma remains largely unclear.

In the present study, we identified a novel lncRNA RP11-284F21.9 that was down-regulated in cervical carcinoma tissue and cell line. We demonstrated that RP11-284F21.9 directly interacted with miR-769-3p as a ceRNA and bioinformatics analysis revealed that miR-769-3p regulated peptidylprolyl isomerase domain and WD repeat-containing protein1 (PPWD1) expression by targeting PPWD1 3′-UTR. We found that RP11-284F21.9-miR-769-3p-PPWD1 axis regulated proliferation, migration and invasion of cervical cancer cells both *in vitro* and *in vivo*. In conclusion, we indicate that RP11-284F21.9 functions as a tumor suppressor in cervical carcinoma via targeting miR-769-3p/PPWD1, providing a potential promising therapeutic target for cervical carcinoma.

## Materials and methods

### Clinical samples

In the present study, cervical carcinoma tissues and the adjacent normal tissues were obtained from the patients underwent surgical treatment at our hospital. All the tissues were stored in liquid nitrogen before RNA isolation. The present study was approved by the Ethics Committee of our hospital. Written informed consent was signed before specimen collection.

### Cell lines and culture

The cervical cancer cell lines Hela, SiHa, C33A, CaSki, and the normal human cervical cell line H8 were obtained from American Type Culture Collection (ATCC). All these cells were maintained in Dulbecco’s modified Eagle’s medium (DMEM) supplemented with 10% fetal bovine serum (FBS) and 1% penicillin–streptomycin. Cells were cultured in a humidified incubator at 37°C with 5% CO_2_. Mycoplasma detection was negative in all cell lines.

### Transfection

Briefly, 3 × 10^5^ cells were seeded in six-well plates. When cells were approximately at 70–80% confluence, the medium was changed to FBS-free DMEM and the transfection was performed using Lipofectamine™ 3000 (Invitrogen) according to manufacturer’s protocols. Cells were grouped as follows: (1) mock control and pcDNA3.1-RP11-284F21.9 group; (2) negative control (NC) group and miR-769-3p mimics group; (3) NC group, pcDNA3.1-RP11-284F21.9 group, miR-769-3p mimics group, miR-769-3p inhibitor group and pcDNA3.1-RP11-284F21.9+miR-769-3p mimics group; (3) NC group, miR-769-3p mimics group, pcDNA3.1-PPWD1 group, miR-769-3p mimics+pcDNA3.1- PPWD1 group. Cells were harvested at indicated time points for further experiments.

### RT-qPCR

Total RNA from tissues and cell lines was extracted by using TRIzol Reagent (Invitrogen). cDNA was synthesized through the PrimeScript RT reagent kit (TaKaRa) according to manufacturer’s instructions. Then real-time PCR assay was performed by employing SYBR PrimeScript™ PLUS RT-PCR Kit (TaKaRa) to detect the expression level of LncRNA-RP11-284F21.9, miR-769-3p and PPWD1. The reaction condition of PCR was 95°C for 30 s, 60°C for 40 s for 40 cycles. β-actin or U6 were used as an endogenous control to normalize. The relative expression levels were counted by 2^−ΔΔ*C*_t_^ method. The primer sequence used in the present study were as follows: LncRNA-RP11-284F21.9: forward, AGGATTGGCACTCACTTCGG, reverse, TCTCTCACCACGTCTGGTCT; miR-769-3p: forward, 5′-GCGGCGGCTGGGATCTCCGGGGTC-3′, reverse, 5′-GTGCAGGGTCCGAGGT-3′; β-actin: forward, 5′-TGTCACCAACTGGGACGATA-3′, reverse, 5′-GGGGTGTTGAAGGTCTCAAA-3′; U6: forward, 5′-ATTGGAACGATACAGAGAAGATT-3′, reverse, 5′-GGAACGCTTCACGAATTTG-3′.

### Cell proliferation assay

After transfection, cell proliferation ability was evaluated by CCK-8 assay (Dojindo). Cells were cultured for 0, 24, 48 or 72 h in 96-well plates, after that, 10 μl of CCK-8 (5 mg/ml) was added to the culture medium in each well. The absorbance at 450 nm was measured by Exl 800 microplate reader (Bio-tek).

EdU assay was performed to determine DNA synthesis in proliferating cells by using an EdU assay kit (Invitrogen). After transfection, cells were cultured for 48 h, fixed with 4% paraformaldehyde and permeabilized by 0.3% Triton X-100. Then cells were incubated with 10 μM EdU for 2 h, and cell nuclei were stained with DAPI (5 μg/ml). The number of EdU-positive cells was counted under a microscope in five random fields (Olympum). All assays were independently performed in triplicate.

### Cell cycle analysis

Briefly, cells were gathered, and fixed with 70% ethanol at 4°C overnight. After that, cells were treated with ribonuclease A (20 μg/ml, Sigma–Aldrich) and incubated with propidium iodide (50 μg/ml, Sigma–Aldrich) for 30 min at 37°C. Then, population in G_2_-M, S and G_0_-G_1_ phases was determined by flow cytometry (Becton Dickinson).

### Wound-healing assay

After transfection, Hela or SiHa cells were seeded in six-well plates and grown to 90% confluence. The wound was created by scratching with a sterile pipette tip (200 μl). The cells were cultured in DMEM with 10% FBS. The closure of wound was monitored by an inverted optical microscope (Olympus) at 0 and 48 h.

### Cell migration/invasion assay

The 24-well transwell chambers (Costar) with Matrigel-coated membranes were used for invasion assay. A total of 2 × 10^5^ Hela or SiHa cells in 100 μl FBS-free DMEM were added to the upper chamber and 500 μl DMEM with 10% FBS were added to the bottom chamber. After 48 h, the invading cells in the bottom chamber were stained with 0.1% Crystal Violet. The cells were observed and calculated under the inverted microscope (Olympus).

### Western blotting

Total protein from tissues or cells was extracted using RIPA lysis buffer with 1% protease inhibitors. BCA protein assay kit (Thermo) was used to determine the concentration of obtained total protein. Thirty microgram of proteins were separated on 10% SDS/PAGE and electro-transferred to PVDF membrane (Millipore). And then were incubated with anti-PPWD1 (Abcam) and anti-β-actin (Abcam) at 4°C overnight. After washing, the membrane was incubated with an HRP-labeled secondary antibody IgG (Abcam) at room temperature for 1 h. Immunolabeling was visualized by using the ECL system (Millipore).

### Luciferase reporter assay

The 3′-UTR of RP11-284F21.9 containing the predicted miR-769-3p binding site was amplified by PCR. And then was cloned into a Dual-luciferase miRNA Target Expression Vector (Promega) for forming RP11-284F21.9-wildtype (RP11-284F21.9-Wt). The same approach was used to forming RP11-284F21.9-mutated-type (RP11-284F21.9-Mut). Similarly, PPWD1-wildtype (PPWD1-Wt) and PPWD1-mutated type (PPWD1-Mut) were set up. Then the luciferase activities were tested by Dual-luciferase reporter assay system (Promega).

### RNA immunoprecipitation assay

RNA immunoprecipitation (RIP) assay was performed by Imprint RNA immunoprecipitation kit (Sigma–Aldrich) according to manufacturer’s instructions. Briefly, IgG-induced Hela cells were collected and resuspended in RIP lysis buffer (Solarbio), subsequently centrifuged at 12000×***g*** for 5 min. Then, cell lysate was incubated with anti-Argonaute2 (anti-Ago2) or anti-IgG (NC) overnight at 4°C, followed by the addition of Protein A magnetic beads to get the immunoprecipitation complex. At last, the relative enrichment of RP11-284F21.9 and miR-769-3p were detected by RT-qPCR.

### Tumor xenograft model

The posterior flank of the 6-week-old male BALB/c nude mice (*n*=10) were subcutaneously injected with SiHa (5 × 10^7^) cells transfected with mock or pcDNA3.1-RP11-284F21.9. Tumor volumes were examined every 4 days, and tumor tissues were photographed and weighed on day 13. At 13 days after inoculation, mice were killed by intraperitoneal injection of tripled 3% pentobarbital sodium. The expression level of miR-769-3p in tumor tissues was detected by qRT-PCR. The protein level of PPWD1 in tumor tissues was measured by Western blot. The expression of Ki-67 was analyzed by immunohistochemical (IHC) staining. All animal work was performed at Xi’an Jiaotong University, and all animal protocols were approved by the ethics committee of the Affiliated Cancer Hospital and Institute of Xi’an Jiaotong University.

### Statistical analysis

Statistical analyses were performed with GraphPad Prism 6.0 software and data were expressed as mean ± SD. Statistical comparisons were made by one-way analysis of variance (ANOVA) and *P*<0.05 indicated a statistically significant difference.

## Results

### RP11-284F21.9 is down-regulated in cervical carcinoma tissues and cell lines

**Figure 1:**
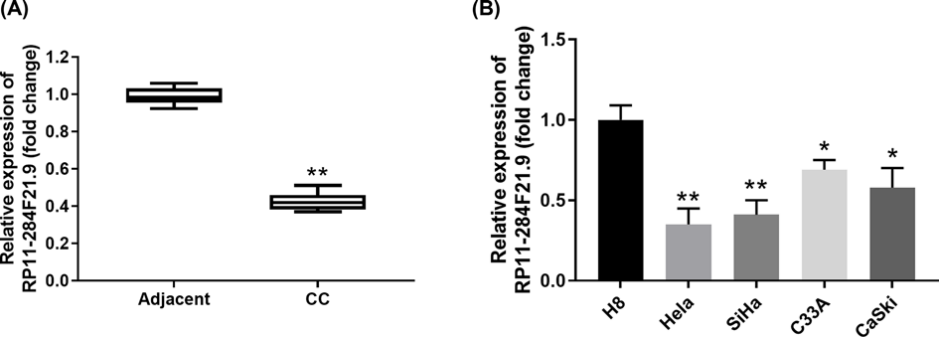
RP11-284F21.9 is down-regulated in cervical carcinoma tissues and cell lines (**A**) Relative expression levels of RP11-284F21.9 in cervical carcinoma tissues compared with that in adjacent normal tissues were analyzed by RT-PCR. (**B**) Relative expression levels of RP11-284F21.9 in human cervical carcinoma cell lines (Hela, SiHa, C33A and CaSki) and normal human cervical cell line H8 was analyzed by RT-PCR. Data were presented as mean ± SD. *P<0.05, **P<0.01.

To investigate the function of RP11-284F21.9 in cervical carcinoma, we first assessed the expression levels of RP11-284F21.9 in cervical carcinoma tissues and adjacent normal tissues. The results showed that RP11-284F21.9 expression was significantly lower in cervical carcinoma tissues in comparison with that in adjacent normal tissues (*P<*0.01, Figure 1A). Furthermore, we demonstrated that the expression levels of RP11-284F21.9 were also remarkably down-regulated in four different cervical cancer cell lines (Hela, SiHa, C33A and CaSki) compared with that in normal human cervical cell line H8 (Figure 1B). Hela and SiHa, which had relative lower RP11-284F21.9 expression, were selected for the subsequent experiments.

### Overexpression of RP11-284F21.9 inhibits proliferation, invasion and migration of cervical carcinoma cells *in vitro*

**Figure 2:**
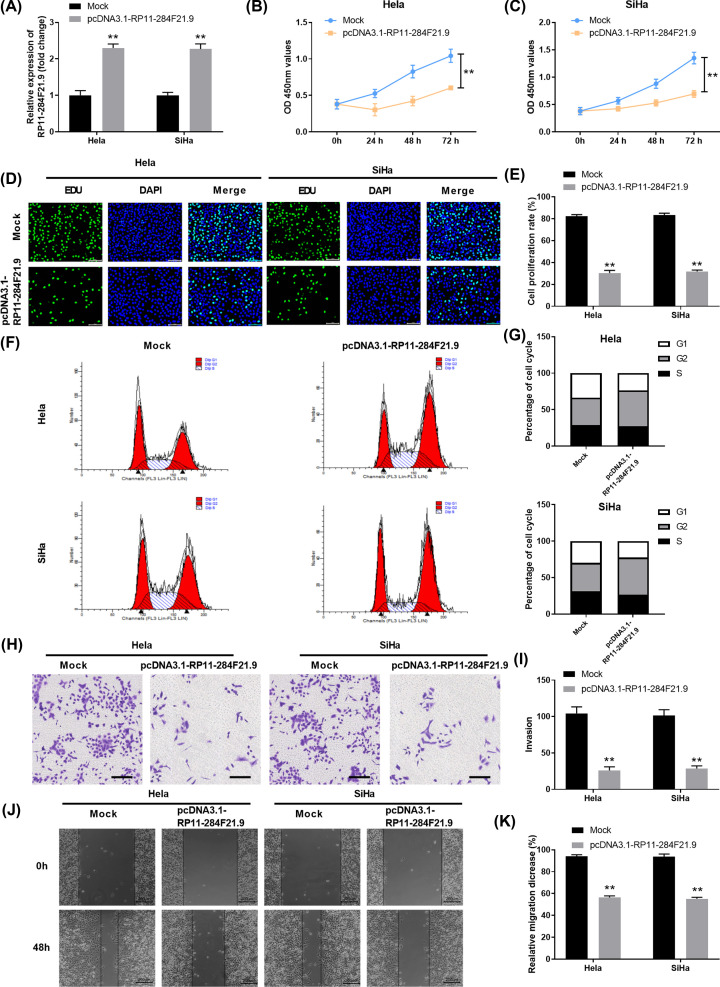
Overexpression of RP11-284F21.9 inhibits proliferation, invasion and migration of cervical carcinoma cells *in vitro* (**A**) Hela or SiHa cells were transfected with mock control or pcDNA3.1-RP11-284F21.9. The overexpression efficiency was examined by RT-PCR. (**B,C**) Cell proliferation of Hela (B) or SiHa (C) cells transfected with mock control or pcDNA3.1-RP11-284F21.9 was assessed by CCK-8 assay at indicated time points. (**D,E**) Cell proliferation of Hela (D) or SiHa (E) cells transfected with mock control or pcDNA3.1-RP11-284F21.9 was assessed by immunofluorescence staining of EdU and DAPI. (**F,G**) Cell cycle analysis of Hela or SiHa cells transfected with mock control or pcDNA3.1-RP11-284F21.9 was analyzed by flow cytometry. (**H,I**) Cell invasion capability of Hela or SiHa cervical carcinoma cells transfected with mock control or pcDNA3.1-RP11-284F21.9 was analyzed by transwell assay. (**J,K**) Wound-healing assay was conducted to determine the relative migration distance of Hela or SiHa cells transfected with mock control or pcDNA3.1-RP11-284F21.9. Data were presented as mean ± SD. **P<0.01.

To evaluate the function of RP11-284F21.9 on cervical carcinoma cells, we constructed pcDNA3.1-RP11-284F21.9 to overexpress RP11-284F21.9. The overexpression efficiency was confirmed in both Hela and SiHa cells (Figure 2A). Cell proliferation assay showed that the proliferation of Hela or SiHa cervical carcinoma cells transfected with pcDNA3.1-RP11-284F21.9 was remarkably decreased in comparison with that in cells transfected with mock control (*P<*0.01, Figure 2B,C). The inhibition of cell proliferation by RP11-284F21.9 overexpression was further confirmed in Hela or SiHa cells by using EdU staining assay (Figure 2D,E). We also performed cell cycle analysis in Hela or SiHa cells transfected with mock control or pcDNA3.1-RP11-284F21.9. Overexpression of RP11-284F21.9 significantly arrested cervical carcinoma cells at G_2_ phase with less cells in G_1_/S phase (Figure 2F,G). Moreover, transwell assay revealed that overexpressing RP11-284F21.9 drastically inhibited cell invasion in Hela or SiHa cervical carcinoma cells (*P<*0.01, Figure 2H,I). In addition, we also conducted wound-healing assay and found that RP11-284F21.9 overexpression decreased the relative migration distance of Hela or SiHa cells (*P<*0.01, Figure 2J,K). Consistently, overexpression of RP11-284F21.9 inhibited the expression of epithelial–mesenchymal transition (EMT) markers and proliferation marker Ki-67 in Hela or SiHa cells (Supplementary Figures S4A and S5A). In summary, our results suggested that RP11-284F21.9 overexpression inhibited proliferation, invasion and migration of cervical carcinoma cells *in vitro*.

### RP11-284F21.9 directly interacts with miR-769-3p and functions as the miR-769-3p sponge

**Figure 3:**
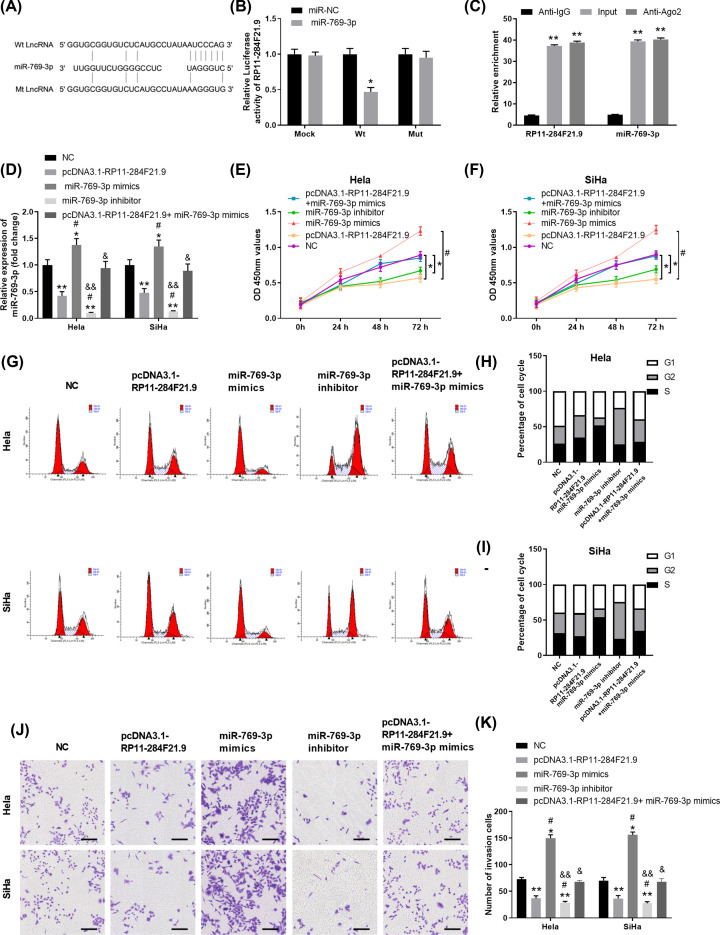
RP11-284F21.9 directly interacts with miR-769-3p and functions as the miR-769-3p sponge (**A**) The potential binding sites between RP11-284F21.9 and miR-769-3p predicted using the DIANA tools. Mutated RP11-284F21.9 binding sites were shown. (**B**) HEK293 cells were transfected with pmirGLO-RP11-284F21.9-WT or pmir-GLO-RP11-284F21.9-Mut, together with miR-769-3p mimic or negative control miR-NC. Seventy-two hours post transfection, the relative luciferase activity was measured. *P<0.05 vs. miR-NC. (**C**) RIP assay was performed using anti-Ago2 or anti-IgG antibodies; the relative enrichment of RP11-284F21.9 or miR-769-3p in Hela cells was analyzed. **P<0.01 vs. Anti-IgG control. (**D**) The expression levels of miR-769-3p in Hela or SiHa cells transfected with NC, pcDNA3.1-RP11-284F21.9, miR-769-3p mimics, miR-769-3p inhibitor or pcDNA3.1-RP11-284F21.9+miR-769-3p mimics were analyzed by RT-PCR 72 h later. *P<0.05, **P<0.01 vs. NC; ^#^*P*<0.05 vs. pcDNA3.1-RP11-284F21.9; ^&^*P*<0.05, ^&&^*P*<0.01 vs. miR-769-3p mimics. (**E,F**) Cell proliferation of Hela (B) or SiHa (C) cells in different groups was assessed by CCK-8 assay at indicated time points. *P<0.05 vs. NC; ^#^*P*<0.05 vs. pcDNA3.1-RP11-284F21.9. (**G**–**I**) Cell cycle of Hela or SiHa cells in different groups was analyzed by flow cytometry. (**J,K**) Cell invasion capability of Hela or SiHa cervical carcinoma cells was analyzed by transwell assay. *P<0.05, **P<0.01 vs. NC; ^#^*P*<0.05 vs. pcDNA3.1-RP11-284F21.9; ^&^*P*<0.05, ^&&^*P*<0.01 vs. miR-769-3p mimics.

To explore the underlying mechanisms that RP11-284F21.9 regulates cervical carcinoma cell proliferation, invasion and migration, we used the DIANA tool combined the dataset of miRNAs involved in cell cycle regulation (GO:0000086) to search for the target of RP11-284F21.9 (Supplementary Figure S1). Emerging evidence has shown that lncRNAs function as ceRNAs in regulating gene expression as miRNA sponges [28,29]. There are 11 miRNAs potentially interacting with RP11-284F21.9 while overexpression of RP11-284F21.9 significantly inhibited the expression of miR-769-3p (Supplementary Figure S1). MiR-769-3p was identified to have the complementary binding sites with RP11-284F21.9 (Figure 3A). Luciferase reporter assay was conducted and the results showed miR-769-3p mimics significantly inhibited the luciferase activity in HEK293 cells transfected with pmirGLO-RP11-284F21.9-WT, but not in HEK293 cells transfected with pmirGLO-RP11-284F21.9-Mut (Figure 3B). We also performed RIP experiment and found that antibody against Ago2 could specifically enrich both RP11-284F21.9 and miR-769-3p (Figure 3C). Pearson correlation analysis revealed that the expression of RP11-284F21.9 was negatively associated with the expression of miR-769-3p (Supplementary Figure S3A). These findings suggested that RP11-284F21.9 could specifically and directly interact with miR-769-3p.

We further verified the interaction between RP11-284F21.9 and miR-769-3p using mi769-3p mimics or inhibitor. As shown in Figure 3D, Hela or SiHa cells transfected with pcDNA3.1-RP11-284F21.9 or miR-769-3p inhibitor significantly inhibited miR-769-3p expression. While miR-769-3p mimics markedly increased the miR-769-3p levels, overexpression of RP11-284F21.9 partially reversed the increase in miR-769-3p. Functionally, compared with that of NC group, overexpression of RP11-284F21.9 or miR-769-3p inhibitor transfection had similar biological functions on Hela and SiHa cells, such as cell proliferation suppression, cell cycle arrested at G_2_ phase and inhibition of cell invasion (Figure 3E–K). MiR-769-3p mimics enhanced cell proliferation and cell invasion, with more cells proceeding to S phase of cell cycle; however, simultaneously overexpression RP11-284F21.9 reversed the function effects of miR-769-3p overexpression (Figure 3E–K). Thus, we demonstrated that RP11-284F21.9 directly interacts with miR-769-3p and functions as the miR-769-3p sponge.

### MiR-769-3p regulates PPWD1 expression by targeting PPWD1 3′-UTR

**Figure 4:**
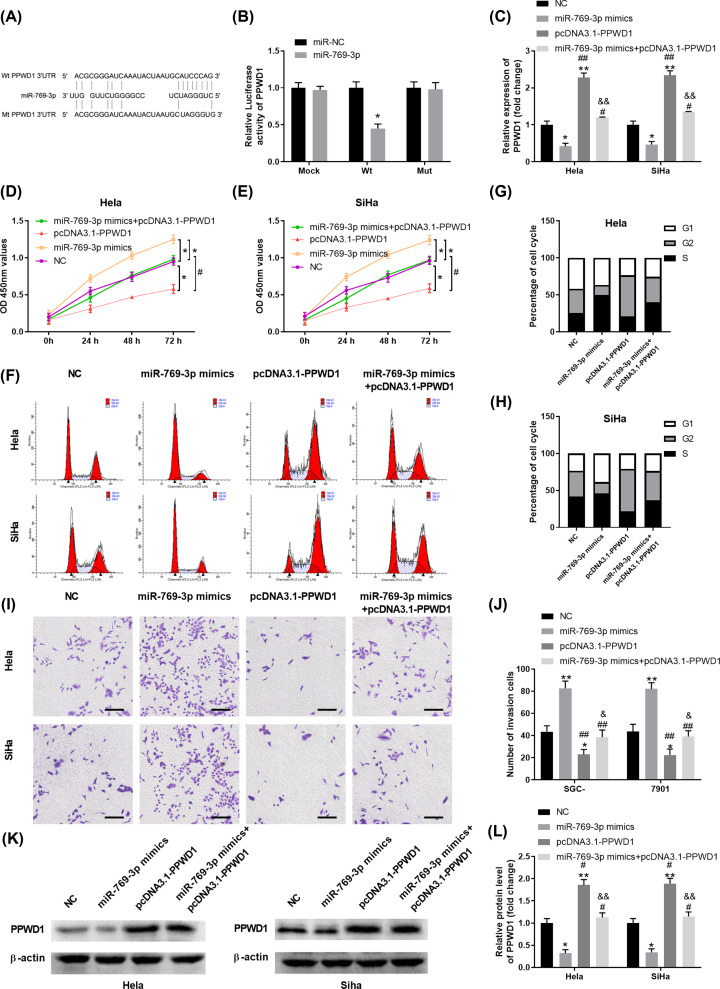
MiR-769-3p regulates PPWD1 expression by targeting PPWD1 3′-UTR (**A**) Diagram of the predicted binding sites of miR-769-3p on the 3′-UTR of PPWD1 and the mutated sequence of 3′-UTR of PPWD1. (**B**) Relative luciferase activity in HEK293 cells transfected with mock control, luciferase reporter vector containing PPWD1 3′-UTR WT sequence (WT) or mutated sequence (Mut), together with miR-NC or miR-769-3p mimics was analyzed. (**C**–**L**) Hela or SiHa cells were transfected with NC, miR-769-3p mimics, pcDNA3.1-PPWD1 or miR-769-3p mimics + pcDNA3.1-PPWD1. (C) The mRNA expression levels of PPWD1 in Hela or SiHa cells were analyzed by qRT-PCR. *P<0.05, **P<0.01 vs. NC; ^##^*P*<0.01 vs. miR-769-3p mimics; ^&&^*P*<0.01 vs. pcDNA3.1-PPWD1. (D,E) Cell proliferation of Hela or SiHa was analyzed by CCK-8 assay. *P<0.05 vs. NC; ^#^*P*<0.05 vs. pcDNA3.1-PPWD1. (F–H) Cell cycle of Hela or SiHa cells in different groups was analyzed by flow cytometry. (I,J) Cell invasion capability of Hela or SiHa cervical carcinoma cells was analyzed by transwell assay. *P<0.05, **P<0.01 vs. NC; ^##^*P*<0.01 vs. miR-769-3p mimics; ^&^*P*<0.05 vs. pcDNA3.1-PPWD1. (K,L) The protein expression levels of PPWD1 in Hela or SiHa cells were analyzed by Western blot. *P<0.05, **P<0.01 vs. NC; ^#^*P*<0.05 vs. miR-769-3p mimics; ^&&^*P*<0.01 vs. pcDNA3.1-PPWD1.

Previous study has shown that miR-769-3p could regulate breast cancer cell apoptosis via down-regulating NDRG1 [30]. To search for the target of miR-769-3p in cervical carcinoma, bioinformatics analysis was performed and PPWD1 was predicted as a direct target of miR-769-3p (Figure 4A). Luciferase reporter experiment confirmed the interaction between miR-769-3p and WT 3′-UTR of PPWD1 (Figure 4B). Consistently, the expression of PPWD1 was significantly down-regulated in cervical cancer tissues and cell lines compared with that in control tissues or cell line (Supplementary Figure S2). Overexpression miR-769-3p inhibited the expression of PPWD1 in Hela or SiHa cells, and transfection with pcDNA3.1-PPWD1 recused PPWD1 expression in Hela or SiHa cells transfected with miR-769-3p mimics (Figure 4C). In addition, Pearson correlation analysis indicated that the expression of miR-769-3p was negatively correlated with the expression of PPWD1 in cervical cancer tissues (Supplementary Figure S3B). We also evaluated the effect of miR-769-3p and PPWD1 overexpression on biological function of Hela and SiHa cells. As shown in Figure 4D–J, miR-769-3p overexpression promoted cell proliferation and invasion, with more cells proceeding to S phase of cell cycle. However, overexpression of PPWD1 had the reverse effects in Hela or SiHa cells. Intriguingly, overexpression of miR-769-3p, together with PPWD1 overexpression in Hela or SiHa cells, resulted in similar cell proliferation, cell cycle distribution and cell invasion compared with those cells transfected with NC (Figure 4D–J). Consistently, miR-769-3p mimics inhibited PPWD1 protein expression while pcDNA3.1-PPWD1 transfection could rescue the protein expression levels of PPWD1 (Figure 4K,L). In addition, overexpression of PPWD1 inhibited the expression of EMT markers and proliferation marker Ki-67 in Hela or SiHa cells, while miR-769-3p mimics exhibited the opposite function (Supplementary Figures S4 and S5). Taken together, our data indicated that miR-769-3p directly regulated PPWD1 expression and miR-769-3p/PPWD1 axis was critical for proliferation and invasion of cervical cancer cells.

### Overexpression of RP11-284F21.9 inhibits tumor growth of cervical carcinoma *in vivo*

**Figure 5:**
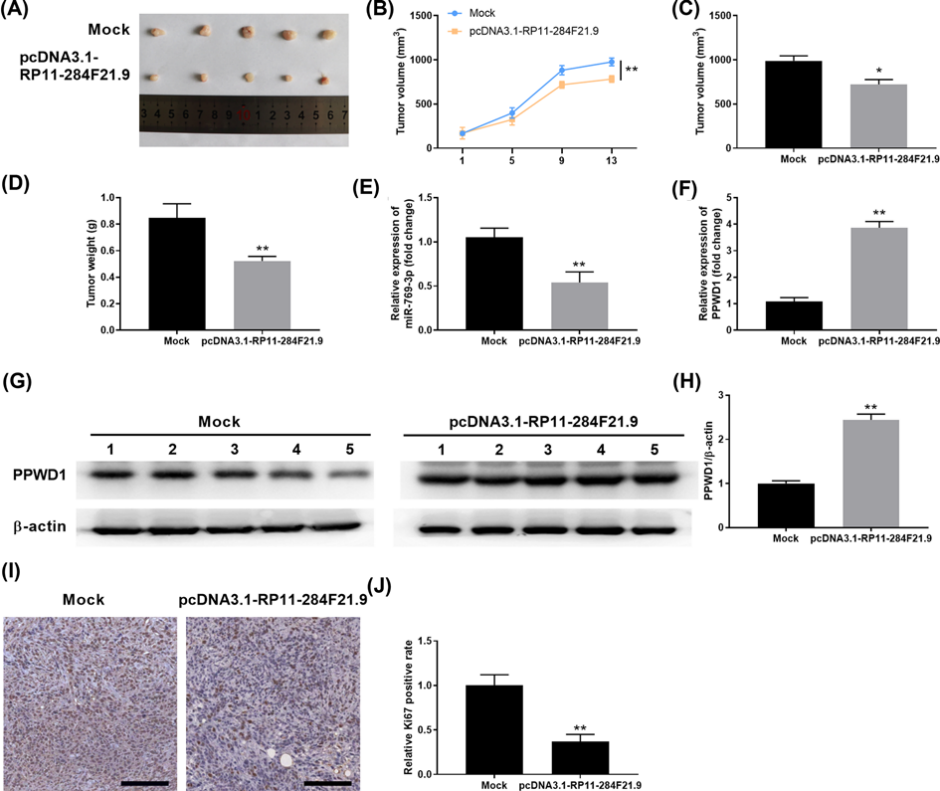
Overexpression of RP11-284F21.9 inhibits tumor growth of cervical carcinoma *in vivo* Cervical carcinoma SiHa cells were transfected with mock or pcDNA3.1-RP11-284F21.9 and then implanted subcutaneously into nude mice to develop tumor. (**A**) Growth curve of tumor volume in nude mice were measured at indicated time points. (**B**–**D**) The volume and weight of tumors from mock or pcDNA3.1-RP11-284F21.9 group were measured at day 13. (**E,F**) The expression levels of miR-769-3p and PPWD1 in the tumor tissues from mock or pcDNA3.1-RP11-284F21.9 group were analyzed by RT-PCR. (**G,H**) The protein expression levels of PPWD1 in the tumor tissues from mock or pcDNA3.1-RP11-284F21.9 group were analyzed by Western blot. (**I,J**) The proliferation marker Ki-67 expression in xenograft tumor tissues was analyzed by IHC staining. **P<0.01 vs. Mock control group.

We further evaluated the function of RP11-284F21.9 on tumor growth *in vivo* using xenograft tumor model. SiHa cells were transfected with mock control or pcDNA3.1-RP11-284F21.9 and implanted into nude mice. Overexpression of RP11-284F21.9 remarkably decreased cervical carcinoma growth and development (Figure 5A). Compared with the tumors in Mock control group, the tumor growth was significantly inhibited in pcDNA3.1-RP11-284F21.9 group with drastically smaller xenograft tumor size/weight (Figure 5B–D). We also checked the miR-769-3p and PPWD1 expression in tumor tissues. As shown in Figure 5E–H, tumor tissues from pcDNA3.1-RP11-284F21.9 group showed remarkably lower expression levels of miR-769-3p, with significantly higher expression levels of PPWD1. Consistently, overexpression of RP11-284F21.9 inhibited cell proliferation, with a lower expression of proliferation marker Ki-67 in xenograft tumor tissues (Figure 5I,J). Overall, our data suggest that overexpression of RP11-284F21.9 inhibits tumor growth of cervical carcinoma *in vivo* via regulating miR-769-3p/PPWD1.

## Discussion

Accumulating evidence has shown that lncRNAs can function as oncogene or tumor suppressors in various cancers [6,31]. LncRNAs can exert their functions as ceRNAs by sponging miRNAs in regulating target mRNAs [21–23]. Here we further extended the understanding of lncRNA–miRNA–mRNA network in cervical cancer by showing that RP11-284F21.9 regulates PPWD1 expressing by competitively binding to miR-769-3p.

LncRNAs show great diagnostic and prognostic values in multiple tumors [32,33]. In this study, we found that lncRNA RP11-284F21.9 was down-regulated in cervical cancer tissues and cell lines (Figure 1), which could be used a novel biomarker for diagnosis and prognosis of cervical cancer. Consistent with our findings, comprehensive network analysis revealed that RP11-284F21.9 was a promising prognostic signature in lung adenocarcinoma [34]. Xiaoshun et. al reported that RP11-284F21.9 could predict lung adenocarcinoma-specific overall survival [35]. Functionally, we also demonstrated that overexpression of RP11-284F21.9 inhibited cervical cancer cell proliferation, invasion and migration *in vitro* (Figure 2) and suppressed cervical tumor development *in vivo* (Figure 5).

MiR-769-3p was predicted to have the complementary binding sequences of RP11-284F21.9 (Figure 3). MiR-769-3p was demonstrated to aberrant expressed in pediatric gliomas by an microRNA microarray assay [36]. Also, miR-769-3p could inhibit cell proliferation and enhance apoptosis in breast cancer MCF-7 cells [30]. We confirmed that miR-769-3p promoted cervical carcinoma Hela and SiHa cells proliferation, migration and invasion in our study. Moreover, overexpression of miR-769-3p could abolish the inhibitory effect of RP11-284F21.9 overexpression (Figure 3). Thus, our results suggest that RP11-284F21.9 and miR-769-3p functionally antagonize each other in cervical cancer cells.

Bioinformatics analysis found that miR-769-3p directly regulated PPWD1 expression by targeting 3′-UTR of PPWD1 (Figure 4). To our knowledge, this is the first report demonstrating the tumor suppressor role of PPWD1 in cervical cancer. Previous studies have shown that PPWD1 might functional relate to cancer pathogenesis as it has the well-characterized WD40 domain which has critical functions in tumorigenesis [37]. Taken together, the regulatory network of RP11-284F21.9-miR-769-3p-PPWD1 could be a reliable drug target for cervical cancer therapy.

## Conclusion

In summary, our findings reveal that lncRNA RP11-284F21.9 might be a tumor suppressor in cervical carcinoma and function as a ceRNA in regulating PPWD1 through competitively binding to miR-769-3p. The RP11-284F21.9/miR-769-3p/PPWD1 axis could serve as a promising prognostic biomarker and therapeutic target for cervical carcinoma.

## Supplementary Material

Supplementary Figures S1-S5

## Data Availability

The datasets used and/or analyzed during the current study available from the corresponding author on reasonable request.

## Abbreviations

Ago2: Argonaute2
CCK-8: Cell Counting Kit-8
ceRNA: competing endogenous RNA
DANCR: Differentiation Antagonizing Non-Protein Coding RNA
DAPI: 4′,6-Diamidino-2-Phenylindole, Dihydrochloride
DMEM: Dulbecco’s modified Eagle’s medium
EdU: 5-Ethynyl-2′-deoxyuridine
EMT: epithelial–mesenchymal transition
FBS: fetal bovine serum
HRP: horseradish peroxidase
lncRNA: long non-coding RNA
MNX1-AS1: MNX1 antisense RNA 1
NC: negative control
PPWD1: peptidylprolyl isomerase domain and WD repeat-containing protein1
RIP: RNA immunoprecipitation
RT-qPCR: quantitative reverse transcription PCR
SNHG14: Small Nucleolar RNA Host Gene 14
SOX21-AS1: SOX21 antisense divergent transcript 1
TUSC8: tumor suppressor candidate 8
VDAC1: voltage-dependent anion channel 1
WT1-AS: WT1 antisense RNA
