# Supplementary material for: Long non-coding RNA RP11-284F21.9 functions as a ceRNA regulating PPWD1 by competitively binding to miR-769-3p in cervical carcinoma
Source: Biosci Rep. 2020 Sep 28;40(9):BSR20200784. doi: 10.1042/BSR20200784 (PMC7527430; doi:10.1042/BSR20200784)
Supplement: Supplementary Figures S1-S5 [file BSR-2020-0784_supp.pdf]

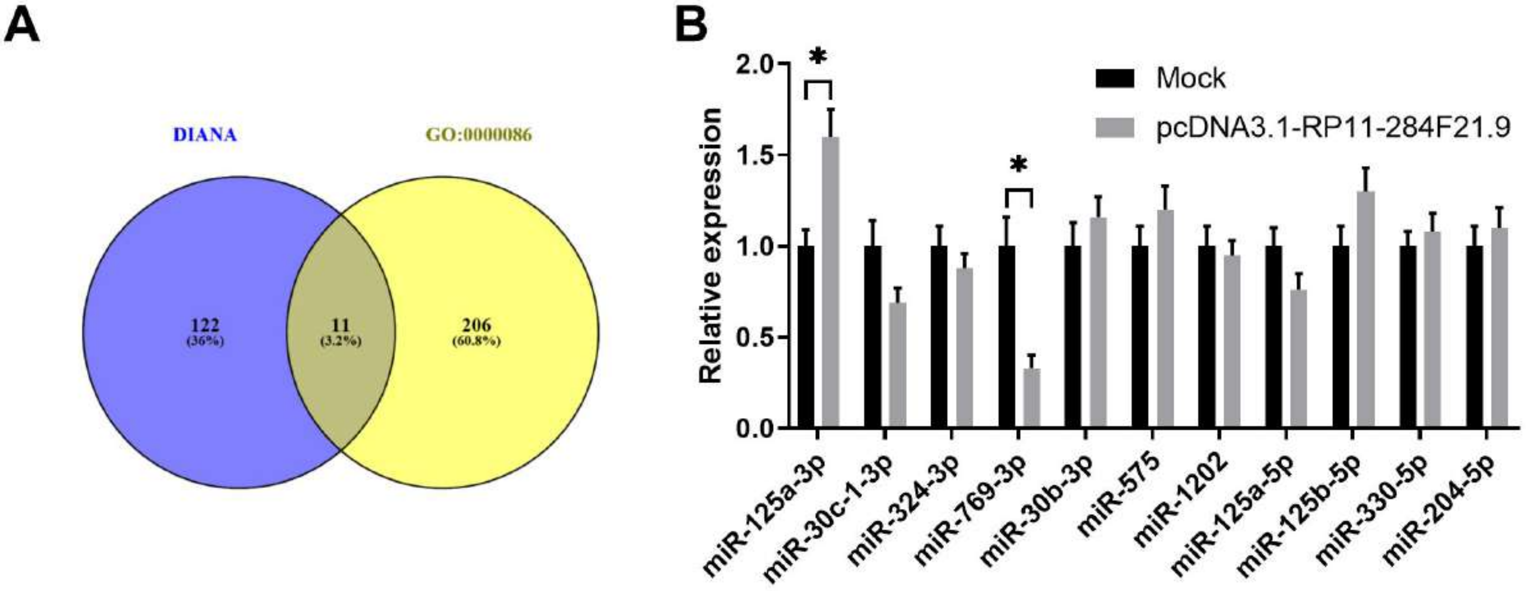
A
DIANA
GO:0000086
122
(36%)
11
(3.2%)
206
(60.8%)
B
Mock
pcDNA3.1-RP11-284F21.9
Relative expression
2.0
1.5
1.0
0.5
0.0
*
*
miR-125a-3p
miR-30c-1-3p
miR-324-3p
miR-769-3p
miR-30b-3p
miR-575
miR-1202
miR-125a-5p
miR-125b-5p
miR-330-5p
miR-204-5p

Figure S1. Bioinformatics analysis predicted RP11-284F21.9 interacted with miR-769-3p. (A) Bioinformatics analysis was performed using DIANA and G2/M transition of mitotic cell cycle|GO:0000086 dataset to predict the potential miRNAs interacting with RP11-284F21.9. (B) The pcDNA3.1-RP11-284F21.9 or mock vector was transfected into HEK293 cells and the expression of 11 miRNAs was analyzed 48 hours later by qPCR. * $p < 0.05$ vs. Mock vector control.

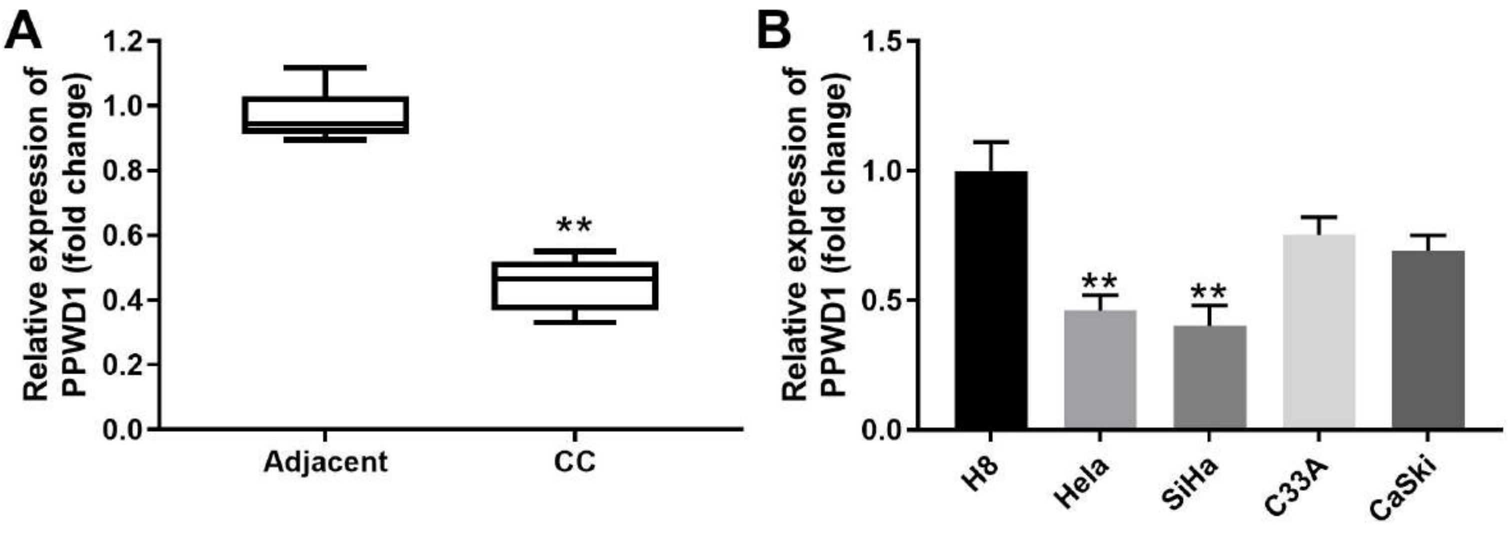
A
Relative expression of PPWD1 (fold change)
1.2
1.0
0.8
0.6
0.4
0.2
0.0
**
Adjacent
CC
B
Relative expression of PPWD1 (fold change)
1.5
1.0
0.5
0.0
**
**
H8
Hela
SiHa
C33A
CaSki

Figure S2. PPWD1 is downregulated in cervical carcinoma tissues and cell lines. (A) Relative expression levels of PPWD1 in cervical carcinoma tissues compared with that in adjacent normal tissues were analyzed by RT-PCR. (B) Relative expression levels of PPWD1 in human cervical carcinoma cell lines (Hela, SiHa, C33A and CaSki) and normal human cervical cell line H8 were analyzed by RT-PCR. Data were presented as mean ± SD. ** $p < 0.01$.

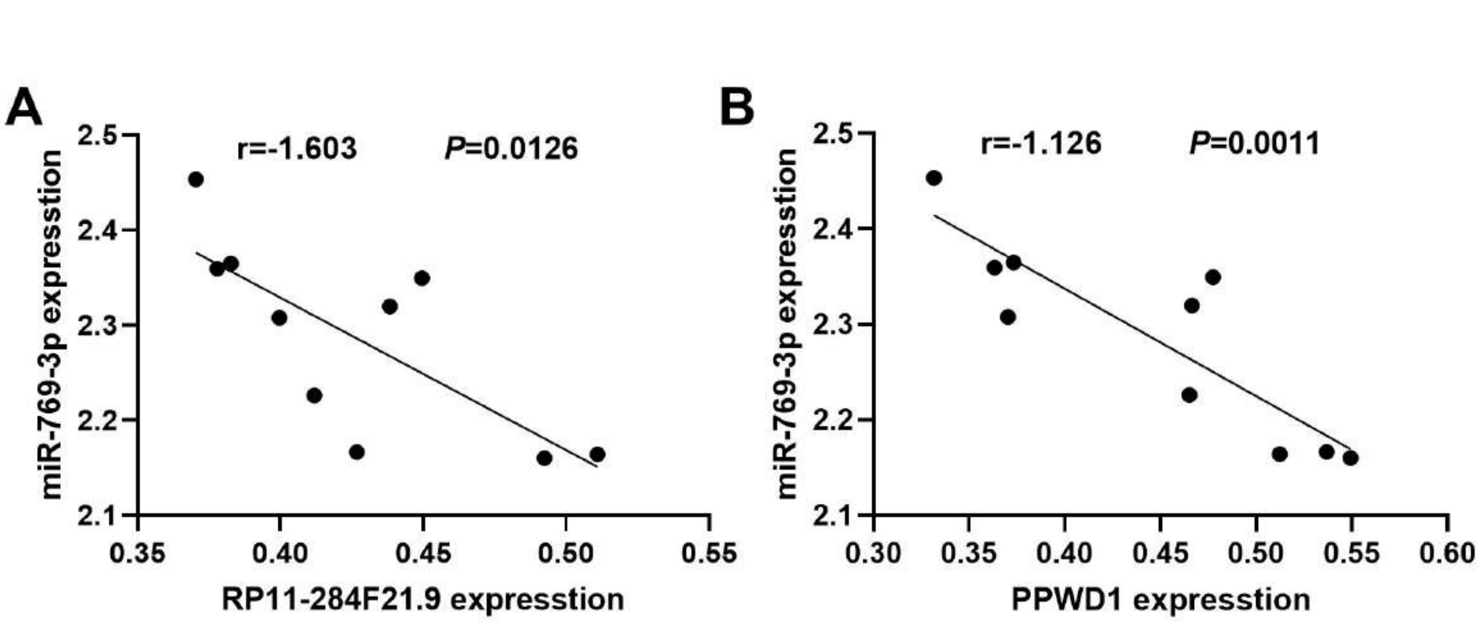
A
r=-1.603
P=0.0126
miR-769-3p expresstion
2.5
2.4
2.3
2.2
2.1
0.35
0.40
0.45
0.50
0.55
RP11-284F21.9 expresstion
B
r=-1.126
P=0.0011
miR-769-3p expresstion
0.30
0.60
PPWD1 expresstion

Figure S3. Pearson correlation analysis between the lncRNA RP11-284F21.9, miR-769-3p, and PPWD1. (A) Pearson correlation analysis was performed between the expression of lncRNA RP11-284F21.9 and miR-769-3p in cervical cancer samples. (B) Pearson correlation analysis was performed between the expression of PPWD1 and miR-769-3p in cervical cancer samples.

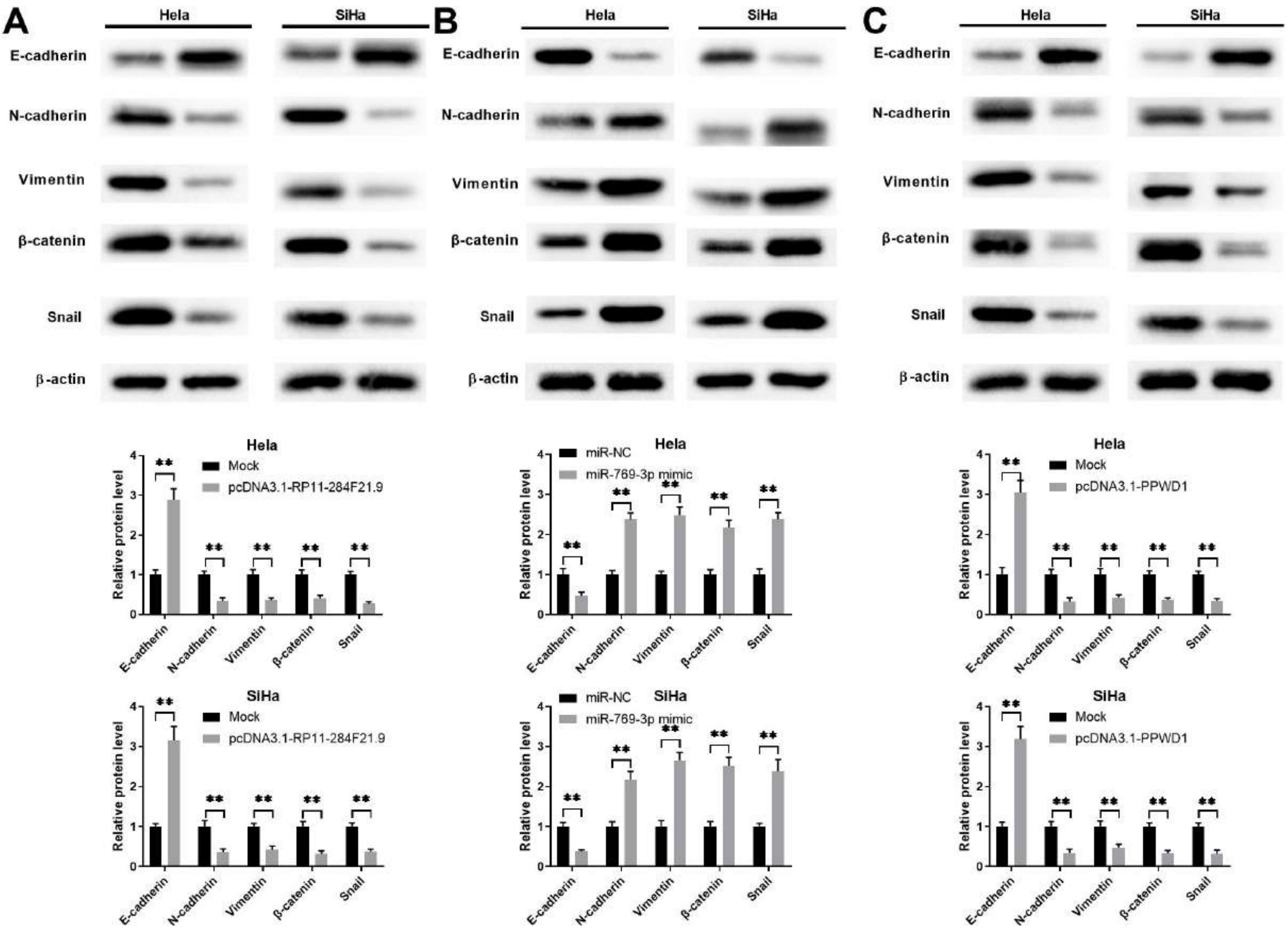

A
B
C
Hela
SiHa
E-cadherin
N-cadherin
Vimentin
β-catenin
Snail
β-actin
Mock
pcDNA3.1-RP11-284F21.9
miR-NC
miR-769-3p mimic
pcDNA3.1-PPWD1
Relative protein level
**

Figure S4. The effect of RP11-284F21.9/miR-769-3p/PPWD1 axis on EMT was evaluated. (A) Hela or SiHa cells were transfected with pcDNA3.1-RP11-284F21.9 or mock vector. The protein expression levels of EMT-related markers E-cadherin, N-cadherin, Vimentin, β-catenin and Snail were analyzed 48 hours later. (B) Hela or SiHa cells were transfected with miR-769-3p mimics or miR-NC. The protein expression levels of EMT-related markers E-cadherin, N-cadherin, Vimentin, β-catenin and Snail were analyzed 48 hours later. (C) Hela or SiHa cells were transfected with pcDNA31.-PPWD1 or mock vector. The protein expression levels of EMT-related markers E-cadherin, N-cadherin, Vimentin, β-catenin and Snail were analyzed 48 hours later. ** $p < 0.01$.

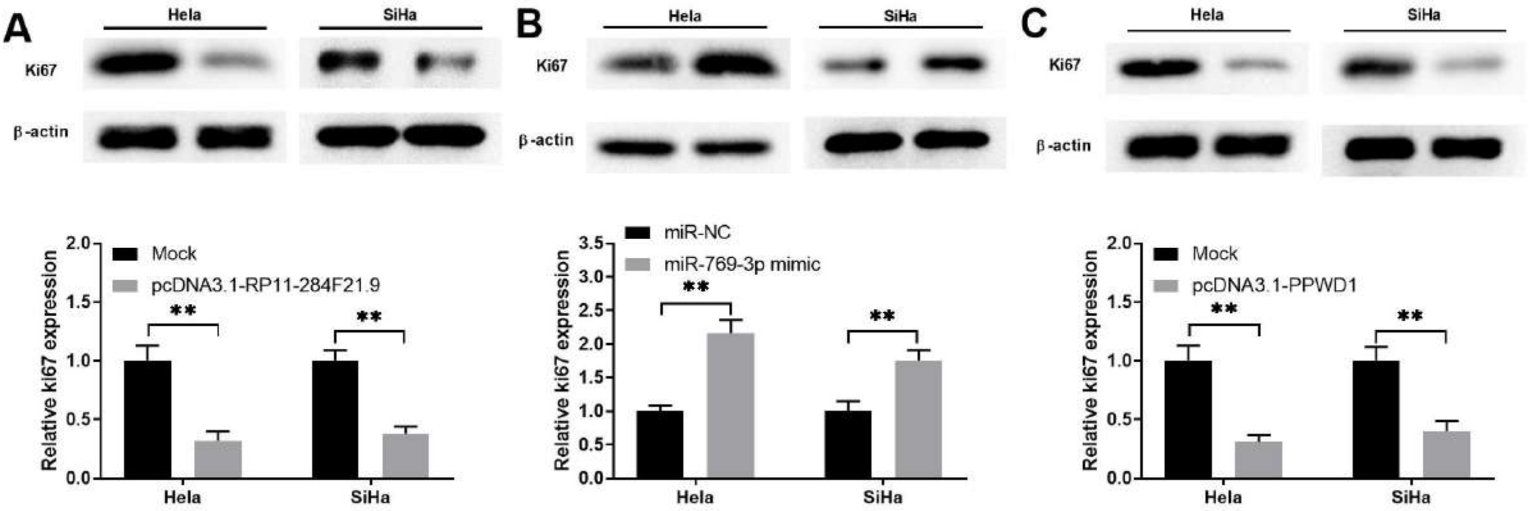
A
Hela
SiHa
Ki67
β-actin
Mock
pcDNA3.1-RP11-284F21.9
Relative ki67 expression
**
B
miR-NC
miR-769-3p mimic
C
pcDNA3.1-PPWD1

Figure S5. The effect of RP11-284F21.9/miR-769-3p/PPWD1 axis on expression of cell proliferation marker Ki-67 was evaluated. (A) Hela or SiHa cells were transfected with pcDNA3.1-RP11-284F21.9 or mock vector. The protein expression levels of Ki-67 were analyzed 48 hours later. (B) Hela or SiHa cells were transfected with miR-769-3p mimics or miR-NC. The protein expression levels of Ki-67 were analyzed 48 hours later. (C) Hela or SiHa cells were transfected with pcDNA31.-PPWD1 or mock vector. The protein expression levels of Ki-67 were analyzed 48 hours later. ** $p < 0.01$.
